# Healthcare in schizophrenia: effectiveness and progress of a redesigned care network

**DOI:** 10.1186/1472-6963-7-129

**Published:** 2007-08-17

**Authors:** Dong-Sheng Tzeng, Li-Chiu Lian, Chin-Un Chang, Chun-Yuh Yang, Gian-Tin Lee, Peter Pan, For-Wey Lung

**Affiliations:** 1 Department of Psychiatry, Military Kaohsiung General Hospital, Kaohsiung, Taiwan; 2 Graduate Institute of Occupational Safety and Health, Kaohsiung Medical University, Kaohsiung, Taiwan; 3 National Health Insurance, Kao-Pin Department, Taiwan; 4 College of Alliance Health, Kaohsiung Medical University, Kaohsiung, Taiwan; 5 Calo Psychiatric Center, Pingdong County, Taiwan; 6 Graduate Institute of Behavioral Sciences, Kaohsiung Medical University, Kaohsiung, Taiwan; 7 Department of Psychiatry, National Defense Medical Center, Taipei, Taiwan

## Abstract

**Background:**

The aim of this study was designed to investigate the care-effectiveness of different healthcare models for schizophrenic patients and the impact of it on caregivers.

**Methods:**

Sample cases were randomly selected from southern Taiwan, 257 patients in redesigned care network, including a general hospital, a chronic ward, 10 outpatient clinics, and multialternative community programs, was compared to 247 patients in other traditional healthcare provider that were utilized as the control group. The quality of life (QOL) questionnaire and the Chinese health questionnaire (CHQ) were used.

**Results:**

The controls had longer duration of illness (*p *= 0.001) and were older (*p *= 0.004). The average resource utilization in the study group (US$ 2737/year, per case) was higher than the control group (US$ 2041) (*t *= 7.91, *p *< 0.001). For the study group, the average length of stay was shorter, but the admission rate was higher. The QOL of the patients in the study group was better than that of the controls (*p *= 0.01). The family burden of the study group was lower (*p *= 0.035) and the score of general health questionnaire higher (*p *= 0.019).

**Conclusion:**

We found that patients in the redesigned care network had a better QOL, lower family burden, decreased days of hospital stay, higher medical resource utilization and less frequent admission to a hospital, and the caregivers had better mental health. Although the costs were higher, the continued care network was more helpful in providing comprehensive mental illness services.

## Background

The impact of deinstitutionalization and the development of community treatment programs for people with serious and chronic mental illness have been frequently discussed for the last 40 years [1,2]. The issue regarding the cost-effectiveness for schizophrenics and caregivers has been given significantly more attention [3-10]. Hence, the costs associated with schizophrenia have become an important issue for Taiwan's Bureau of National Health Insurance (BNHI) [11]. The Kao-Ping Branch of the BNHI in Taiwan calculated the annual medical cost of schizophrenia in 2001 as 4.16% of the total medical budget (US$ 22,928,522/US$ 551,051,725). Therefore, apart from analyzing the utilization of medical resources among providers, the BNHI has also focused on an evaluation and comparison of medical outcomes.

Mental health services in Taiwan are determined partly by the BNHI, which makes overall plans for the national health insurance program, which is a social insurance structure. BNHI utilizes a fee-for-service reimbursement policy. Publicly and privately owned providers of mental healthcare services include general clinics, psychiatric wards of general hospitals, specialized psychiatric hospitals, chronic psychiatric hospitals, psychiatric rehabilitation institutions, day hospital admissions, and home visit services. Treatment plans among different organizations are similar but inconsistent, and lack a complete health network. There were a few patient referrals between organizations. Consequently, patients presented with minor problems at acute care hospitals, thus overcrowding hospitals and compromising the quality of care. Although a registration fee and a co-payment system apply to consultations in general hospitals, medication costs and examination co-payments continue to rise. However, schizophrenia is considered a severe disability under the BNHI guidelines, and patients are entitled to a severe disability card, which allows them to access medical services free of charge at any institution. This system not only increases accessibility to medical services for patients and their families, but also increases the utilization of medical resources. The BNHI has expressed concern over this situation, and has encouraged the establishment of a health network among health service organizations, to provide care to patients in a most cost-effective way.

This study was designed to compare the cost-effectiveness of an integrated treatment model consisting of a teaching hospital, psychiatric rehabilitation institutions, a day hospital, community rehabilitation centers, daycare farms, home-based care, and outpatient clinics, with those of the traditional treatment model provided by psychiatric hospitals or general acute care hospitals under the jurisdiction of the Kao-Ping Branch of the BNHI. We determined the differences in psychiatric resource utilization between these two treatment models with distinct management goals. The treatment outcomes of the two service providers were also explored in this study.

## Methods

### Participants

The patients of this study were recruited from Southern Taiwan, consisted of 371 persons who randomly selected by Alphabetic order from 1210 schizophrenic patients from the redesigned care network model. The control group consisted of 730 persons randomly selected by Alphabetic order from 18,911 schizophrenic patients who did not fall under the above-mentioned network of treatment organizations.

#### A network system of mental health care in the Kao-Ping area

A network of mental health services was created by coordinating a general acute care hospital, a day hospital, a psychiatric rehabilitation institution, a community rehabilitation center, home visit providers, a specialized psychiatric hospital, and 10 local clinics. To provide continued care, 1,210 severely schizophrenic patients, or 1/13 of the schizophrenic patients in Kao-Ping county, were assessed and placed in a different mental care facility, based on the clinical pathway of the treatment model operating at the particular health service organization. Patients were referred to appropriate organizations and with flexibility in timing, place, provider, service, and cost principles.

### Research materials

#### The Taiwanese concise version of the quality of life questionnaire of the world health organization (WHO-QOL-BREF)

The World Health Organization Quality of Life [12-14] (WHOQOL) is a 100-item generic measure designed for use with individual quality of life and mental health. The Taiwan version of WHOQOL was modified and simplified by Yao [15]. Apart from simplifying and translating the questionnaire into Chinese, culturally relevant additional questions of respect and food aspect were included to form a self-completing questionnaire consisting of 28 questions with a 5-point scale. The questionnaire evaluates the objective quality of life of the participants over the past 2 weeks regarding four main aspects: physiological health, psychological health, social relations, and environmental dimension. Higher scores correspond to a better quality of life. Cronbach's Alpha was 0.68 to 0.77 and test-retest reliability was 0.41 to 0.79 [15].

#### Caregiver's burden questionnaire

This questionnaire consisted of 18 questions to assess the burden of the caregivers. The questionnaire looked at problems and emotions encountered by caregivers during the care of a schizophrenic patient, and included two major categories of objective and subjective burdens. Five subcategories were also examined: There were seven questions on "family interference," a subjective burden; the objective burden included two questions on "stigmatism," two questions on "guilt," three questions on the "anxiety state of the caregiver," and two questions on "patient dependency." The responses are in 5-point Likert-type scale. Each item was scored from 0 (never) to 5 (almost always), and the scores were added up in total to reflect the burden on the caregiver. Higher scores represent heavier burdens [16,17]. Overall, the internal consistency for the questionnaire was 0.88, and for each individual item, 0.65–0.90. The reliability of the questionnaire was 0.90, and the correlation coefficient between each individual item and its subcategory was 0.45 [16,17].

#### Chinese health questionnaire (CHQ)

Cheng and Williams developed the CHQ in 1986 [18], with reference to the General Health Questionnaire designed by Goldberg and supplemented by some culturally relevant questions. There are 12 questions with a weighted classification, sensitivity, and specificity of 89%, 70%, and 95%, respectively [19]. This questionnaire is a self-assessed screening instrument used to assess psychiatric morbidity, with a 4-point scale system with options of "not a bit," "as usual," "slightly more than usual," and "much more than usual." The CHQ can be applied effectively in clinical and community settings. Its factor structure consistency is high and Cronbach's α coefficient for internal consistency is between 0.83 and 0.92 [20]. This questionnaire is suitable as a screening assessment for the caregiver's state of psychological well-being.

### Data collection

This study was approved by the hospital ethics committee, which serves as the institutional review board. Starting in September 2002, consent forms and a detailed study description were sent to randomly selected patients and their families by the Kao-Ping Branch of the BNHI. Postage-paid return envelopes were enclosed for the convenience of the patients and for data collection. After obtaining consent from patients and their main caregiver to participate in the study, questionnaires were sent to the patients and their families. The questionnaire recorded variables including age, sex, duration of the disease, and quality of life of the patient, and sex, age, marital status, annual income, CHQ score, and burden score of the main caregiver. If for any reason the questionnaires were not completed, a senior and trained interviewer from the Department of Health or this study team would pay a home visit to help subjects complete the questionnaire. Participants were asked to fill up the same questionnaires 6 months later, as well.

### Statistical analysis

This study used SPSS statistical software version 10.1 [21] to perform descriptive statistics and exploratory data analyses, to determine whether independent variables were normally distributed. A *t *test was used to compare the results of the patient's quality of life, burden on the family and caregivers, and CHQ scores.

## Results

### Baseline demographics

In the first phase of study, there were 257 out of 371 patients turned in their signed consent form. Seventy-six patients refused to participate in the study. Thirty-two patients had an incorrect mailing address. Four patients had passed away, one was in police custody, and one had an amended diagnosis. The control group consisted of 730 people randomly selected by a computer at the BNHI. After two reminder mailings, 241 valid questionnaires returned, 476 patients refused to participate in the study, 11 patients had passed away, one was in police custody, and one had an amended diagnosis.

In the second phase, questionnaires were sent only to those who returned a valid questionnaire in the first phase. The study group returned 190 copies of valid questionnaires, 51 patients refused to participate, and 16 with incorrect mailing addresses. The control group returned 133 copies of valid questionnaires, 86 patients refused participation, 21 had an incorrect mailing address, and one patient had died. Within the sampling population, males predominated among those who refused to participate in both groups, in both the first and second phases of the study.

With regard to demographic data, seven variables were examined: the patient's sex, age, duration of the illness, and the caregiver's sex, age, marital status, and annual income. There were no significant differences in the demographic data except that the patients in the control group had a higher average age (*p *= 0.001) and longer duration of the disease (*p *= 0.004) (Table 1).

**Table 1:** Demographic data of the two groups of schizophrenic patients and their caregivers

**Patient**		**Study group (n = 257)**	**Controls (n = 241)**	**χ^2^**	***p***
Sex	Male	171	146	3.07	0.080
	Female	79	94		
	Data missing	7	7		

		**Study group**	**Controls**	***t*-test**	***p***

Age (years)		39.2	42.8	-3.45	0.001
Duration of disease (years)		13.6	16.1	-2.86	0.004
Hospital admission rate (%)		1.18	10.08		

**Caregiver**		**Study group (n = 257)**	**Controls (n = 247)**	***t*-test**	***p***

Age (years)		56.9	57.5	-0.51	0.61

		**Study group**	**Controls**	**χ^2^**	***p***

Sex	Male	122	120	0.28	0.597
	Female	132	118		
	Data missing	3	9		
Marital status	Married	197	171	6.13	.152
	Single	24	24		
	Others	36	52		
Annual income	<US$ 7500	153	130	7.16	0.061
	US$ 7500–15,000	67	58		
	US$ 15,000–22,500	18	27		
	>US$ 22,500	11	20		

US$ 1 = NT$ 32

### Resource utilization

An analysis of the psychiatric resource utilization for 20,121 schizophrenic patients in this area showed an average cost of US$ 2083 per patient per year in the Kao-Ping area (data not shown). Frequencies of visits for the two groups are shown in Table 2. As shown in Table 3, clinical consultation costs were higher in the control group than in the study group (*p *< 0.001). In terms of home nursing care, almost every patient in the study group had had home visits by healthcare staff, at an average of 2.9 times per year, which was significantly more than the control group (*p *< 0.001); these home visits were associated with higher costs in the study group than in the control group (*p *< 0.001). Regarding the length and number of acute hospital admissions, both were higher in the study group than in the control group (*p *< 0.001). The average costs per admission were similar between the two groups (*p *= 0.08). The study group had a significantly shorter length of chronic hospital admission (160 days) than the control group (210 days; *p *< 0.001); the average cost per admission was higher in the control group (*p *< 0.001), and the number of admissions was higher in the study group (*p *< 0.001). The average number of days recorded for day admission was similar in both groups (*p *= 0.192), but cost per admission was higher in the study group than in the control group (*p *< 0.001). In terms of the psychiatric rehabilitation institutions, average cost per admission and number of admissions were both higher in the control group than in the study group (*p *< 0.001). Total medical expenses per admission at the above-mentioned organizations were US$ 2319 for the study group and US$ 1467 for the control group (*p *< 0.001). Average total medical costs per year were US$ 2737 for the study group and US$ 2041 for the control group. Drug costs accounted for 14.6% of the total costs in the study group and 25.9% in the control group. Drug costs for the Kao-Ping area accounted for 25% of the total medical expenses (Table 3).

**Table 2:** Frequency of visits for the two groups

**Items**	**Study group (*N *= 1210)**	**Control group (*N *= 18911)**	***t***	***p***
Frequency of clinic visits (no. of patients involved)	12.4 (1047)	12.2 (13417)	0.795	0.426
Frequency of home-based care (no. of patients involved)	2.9 (1210)	0.4 (14875)	36.22	<0.001
Community rehabilitation (days)	0.06	1.35	-2.97	0.017
Frequency of community rehabilitation (no. of patients involved)	0.18 (1210)	0.05 (14875)	6.14	<0.001
Acute hospital admission (in days)	77	64	4.865	<0.001
Frequency of acute hospital admissions (no. of patients involved)	2.83 (587)	1.4 (3005)	27.15	<0.001
Chronic hospital admission (in days)	159.6	209.5	-5.39	<0.001
Frequency of chronic hospital admissions (no. of patients involved)	1.52 (212)	1.32 (2366)	5.08	<0.001
Day admission center (in days)	141.7	157.4	-1.306	0.192
Frequency of day admission center usage (no. of patients involved)	2.10 (117)	1.21 (565)	12.02	<0.001
Frequency of psychiatric rehabilitation institution visits (no. of patients involved)	4.92 (72)	7.88 (234)	-5.34	<0.001

**Table 3:** Various associated costs for the two groups

**Items (US$)**	**Study group (*N *= 1210)**	**Control group (*N *= 18911)**	***t***	***p***
Clinic-total cost	485	636	-7.06	<0.001
Clinic-medication cost	244	403	-10.29	<0.001
Clinic-psychiatric treatment cost	26	28	-1.13	0.258
Home-based care – total cost	49	11	19.87	<0.001
Community rehabilitation – total costs	18	8	3.125	0.002
Acute hospital admission – total cost	3485	3253	1.74	0.081
Chronic hospital admission – total cost	3878	5353	-6.622	<0.001
Day admission center-total costs	2593	3436	-3.389	0.001
Psychiatric rehabilitation institution – total cost	317	1447	-11.24	<0.001
Total hospital admission costs	2319	1467	9.382	<0.001
Total hospital drug costs	166	149	1.424	0.155
Total hospital psychiatric treatment costs	503	169	21.76	<0.001
Average cost per patient	2737	2041	7.91	<0.001
Total medical cost	3,314,455	38,593,805		

Currency conversion: US$: NT$ = 1: 32

The results showed improvements in the patients' physiological and psychological health, social relations, environment, and quality of life in the study group. However, higher CHQ scores also meant a decline in the health of the caregivers in the study group (*p *< 0.001). The decrease in the caregiver's burden was statistically significant in the study group (*p *< 0.001). The results are shown in Table 4. Conversely, physiological (*p *= 0.007) and social relations (*p *= 0.043) have statistically significant decrease in the control group. Although the caregiver's burden was not statistically significant in the controls (*p *= 0.882), higher CHQ scores were shown in the 2nd test for the controls (*p *< 0.001). The results are shown in Table 5.

**Table 4:** Comparison between the 1st and 2nd tests for the study group (*N *= 190)

	**1st test Mean ± SD**	**2nd test Mean ± SD**	**Comparison**		**Correlation**	
			
			***t***	***p *value**	***r***	***p *value**
**Patient quality of life questionnaire**						
Physiological health	20.90 ± 4.98	21.08 ± 4.51	-0.39	0.69	0.53	0.47
Psychological health	16.69 ± 4.23	17.27 ± 3.88	-1.47	0.14	0.10	0.16
Social relations	7.42 ± 2.67	7.58 ± 2.77	-0.58	0.56	0.06	0.43
Environment	23.06 ± 5.46	23.89 ± 5.07	-1.58	0.12	0.07	0.37
**Main caregiver**						
Burden load	28.11 ± 9.13	24.14 ± 8.77	4.39	0.00**	0.03	0.65
CHQ	14.44 ± 2.57	18.39 ± 5.02	-9.82	0.00**	0.03	0.64

**Table 5:** Comparison between the 1st and 2nd tests for the control group (*N *= 133)

	**1st test Mean ± SD**	**2nd test Mean ± SD**	**Comparison**		**Correlation**	
			
			***t***	***p *Value**	***r***	***p *Value**
**Patient quality of life questionnaire**						
Physiological health	21.44 ± 5.09	19.67 ± 5.64	2.75	0.01**	0.04	0.67
Psychological health	16.99 ± 4.52	16.05 ± 4.89	1.76	0.08	0.13	0.15
Social relations	8.02 ± 2.66	7.39 ± 2.66	2.04	0.04*	0.14	0.11
Environment	23.67 ± 5.78	22.23 ± 6.65	1.93	0.06	0.04	0.61
**Main caregiver**						
Burden load	26.77 ± 9.03	26.57 ± 11.78	0.15	0.88	-0.13	0.13
CHQ	13.87 ± 2.40	17.01 ± 5.43	-5.72	0.00**	-0.15	0.09

Comparing outcome variables between the two groups, the results showed that patients in the study group had better physical health outcomes than the control group in the 1st test (*p *= 0.02) and the 2nd test (*p *= 0.01). The psychological health of the study group was better than that of the control group in the 2nd test (*p *= 0.01). The environmental health of the study group was better than that of the control group in the 2nd test (*p *= 0.01); and the total QOL score of the study group was higher than that of the control group in the 2nd test (*p *= 0.01). The burden on caregivers in the study group in the 2nd test was lower than that in the control group (*p *= 0.035). For the CHQ scores of caregivers, the 2nd test of the study group was better than that of the control group (Table 6).

**Table 6:** Comparison between the study group and the control group

	**Study group (N1/N2 = 257/190)**	**Control group (N1/N2 = 241/133)**	***t***	***p***
**Patient quality of life questionnaire**				

Physiological health:				
1st test	23.10	20.23	-2.23	0.02
2nd test	21.09	19.67	-2.52	0.01
Psychological health:				
1st test	17.02	16.36	-1.68	0.11
2nd test	17.27	16.05	-2.51	0.01
Social relations:				
1st test	7.36	7.76	1.64	0.88
2nd test	7.58	7.39	-0.61	0.54
Environment:				
1st test	23.37	22.93	-0.86	0.39
2nd test	23.89	22.23	-2.55	0.01
Total score:				
1st test	81.32	78.93	-1.47	0.14
2nd test	81.75	76.56	-2.55	0.01

**Main caregiver**				

Burden load:				
1st test	27.25	27.76	0.62	0.53
2nd test	24.14	26.57	2.12	0.035
CHQ:				
1st test	14.30	14.36	0.27	0.79
2nd test	18.39	17.01	-2.35	0.019

## Discussion

The community-based network system of the current study provides a full service to help psychiatric patients develop skills for coping with the problem of living in the community and which virtually decreases hospitalization. Different to traditional hospital basis, community-based multidisciplinary teams are not only responsible for individual caseloads (case management) by homecare member, but also provide linking and coordinating services by a network system, that with a variety of community resources. This model shows how patients who would be treated in mental hospital can be successfully treated in the community without shifting the burden of care to their families. Despite the increasing costs of medication reimbursement, and the increased use of the community mobile team and outpatient clinic, the overall costs for the mental health system were substantially reduced.

The direct costs of care were found to be much lower than the costs in European countries. Average costs in Taiwan were US$ 2084 (US$ 2737 for the study group and US$ 2041 for the control group individually). Other countries reported the following associated costs: US$ 2693 in Spain [22], US$ 15,859 in Mannheim, Germany [23], US$ 32,003 in West Lambeth, England (which includes informal care costs) [24], US$ 5678 in Italy [25], and US$ 7656 in South Verona, Italy [26]. When compared with USA, there is 1.56 times as much cost as that in Taiwan, with adjusted per capita income of the two countries. It shows that the average costs in Taiwan are further less than the costs in USA. In our study, the inpatient costs for the study group were 78% (acute 55.2%, chronic 22.2%) of the total health costs, and 66% (acute 30.17%, chronic 36.2%) for the control group; these costs seemed to dominate the overall service costs. Costs varied from 38% to 93%, and were variable internationally [27]. Drug costs as a percent overall healthcare cost in our study were much higher than the drug costs in other counties. The average drug costs in this study were 25% of total direct healthcare costs, 14.6% in the study group, and 25.9% in the control group, when compared with the cost in other countries, which ranged from 2.3% to 13% [27]. In an extended study of 10,972 cases across more than 10 countries in Europe, most patients were prescribed atypical neuroleptics at their first medical consultation [22]. In all, a variation was found between the two groups in our study, and an evident variation was also found in European countries. Drug costs are expected to be proportionately higher in developing countries, compared with developed countries [28]. Further evaluations are needed regarding the cause of the lower drug costs in the study group, the higher percentage of drug costs in Taiwan, and the percentage of prescriptions of atypical neuroleptics.

Many studies have discussed the reasons associated with the costs of schizophrenia. Compatible with previous findings, we found that the average length of stay (77 days) in the study group was longer than in the control group (64 days), and the average age of patients in the study group was 39.2 years, younger than the control group at 42.8 years old. The readmission rate in the acute ward of the study group (2.83 in 1 year) was much higher than in the control group (1.4 in 1 year), which might be why the direct costs of the study group were higher than those of the control group. The high readmission rate might suggest severe mental symptoms, lower GAF scores, higher BPRS scores, and lower income [29]. According to the findings of this study (Tables 1 and 2), the higher costs of the study group might be due to the clinical differences of the cases.

The report by Knapp suggested that some costs were very difficult to determine. Although overall direct and indirect costs in the Knapp report added up to 2.6 billion euros, some indirect impacts were not measured, thus making the estimation of indirect costs a difficult task. According to the report published by the Department of Health, UK [30], the costs for schizophrenia treatment were 5.4% of the total medical budget, and 3% of the total expenditure by the National Health Service [31]. Generally, the direct costs of schizophrenia could be expected to be from 1.5% to 3% of the total national healthcare expenditure [32-34]. In our study, the higher resource utilization of schizophrenic patients was 4.12% of the total healthcare resources of the Kao-Ping area, which might be due to Taiwan's social reform in recent years, in which the policymakers encouraged the development of mental health services that would elevate hospital ranking.

With respect to medical outcomes, we found that the following factors are often associated with a lower quality of life: sex (female), multiple episodes of schizophrenic psychosis, longer duration of the disease, and severe psychotic symptoms, such as with patients with higher BPRS scores [35]. One study reported no differences in subgroups in terms of service utilization, quality of life, and the caregiver's emotional satisfaction between male and female patients [36]. Many patients in Italy lived at home, with a hospital admission rate of 5.5%. They had a better quality of life when compared with patients in USA (with a hospital admission rate of 21%), because living at home meant greater residential stability, more ownership, and more people contacts [37]. In this study, the poorer quality of life in the control group might be due to longer duration of hospital admissions and less home visiting. According to this study, the mental health provider should set up alternative services for the individual needs of patients and families of different genders, ages, and family incomes.

With regard to the families, our previous study revealed that an older age, a shorter duration of the disease, and severe psychotic symptoms were the predictors of a heavier burden on families [35]. In this study, the factors that influenced the caregivers' burden were the longer duration of the disease and the low income of the caregivers. Further evaluation is needed regarding whether a different case sample, factor of poverty, demographic distribution, or interventions such as a study group, which have a higher service contact rate, would lower the family burden. In another study, psychological health and the burden on families were significantly associated with a shorter duration of the disease, showing that care giving created a burden on the family [38]. In a study by the Connecticut Health Care System of the USA, patients with severe psychotic symptoms, living in the community, and with frequent family contact were correlated to a heavier family burden [39]. Regardless of the type of service the patient received, the acute onset, severe symptoms, and frequent contact with the family were usually associated with a greater burden and mental stress. In the network of services in the study group, the continued follow-up of each patient may have been a better approach to decrease the family burden.

Inconsistencies were observed in family burden and CHQ scores, which may be due to the fact that family burden was more related to providing care for the patient, while the psychological health of the family was more related to secular changes. The CHQ scores were influenced by the age of the caregivers: the older they were, the lower the CHQ score (*p *= 0.045, table not shown). Regardless of the type of care system, the caregivers were constantly under enormous psychological stress. As time passed, the CHQ scores of the caregivers increased, especially among the younger caregivers, which should be taken into consideration when using the CHQ as an outcome variable. The outcome variance due to multiple informants, such as that of the CHQ of the caregivers, cannot be ignored. Even self-rated satisfaction reports completed by parents are better predictors of the patient's satisfaction, and are more sensitive than changes in the patient's psychotic symptoms [40,41]. This indicates that the psychological status of the caregiver and the family should be further investigated when promoting the healthcare management of schizophrenia.

## Conclusion

Indeed, the cost comparison analysis would be more convincing if it was beyond the small number of participants (257 study patients vs. 247 control patients), however, we could only get the costs of two larger populations from the government. This might be a limitation for generalization in this study. In conclusion, we found that the costs of the study group were higher than those of the control group, but the QOL of the study group was higher and the family burden lower than that of the control group. Moreover, most patients of the study group were frequently admitted to a hospital but with decreased length of hospital stay. At a time of scarce medical resources with many countries paying close attention to disease management, the promotion of the community-based treatment model should include the consideration of individual factors such as the patient's sex, age, duration of the disease, social functioning, and the family support system. Treatment models should be tailored to individual needs [42]. The findings from studies of these models is an informative reference for future designations of community mental health service programs in Taiwan.

## Competing interests

The author(s) declare that they have no competing interests.

## Authors' contributions

Study concept and design: FWL.

 Acquisition of data: DST, LCL, CUC, CYY, GTL, PP.

Analysis and interpretation of data: DST, LCL, CUC, CYY, GTL, PP, FWL.

Drafting of the manuscript: DST, FWL.

Critical revision of the manuscript for important intellectual content: DST, FWL.

Statistical analysis: DST, FWL.

Obtained funding: DST, FWL.

Administrative, technical, or material support: FWL.

Study supervision: FWL; FWL had full access to all the data in the study and takes responsibility for the integrity of the data and the accuracy of the data analysis.

All authors read and approved the final manuscript.

## Pre-publication history

The pre-publication history for this paper can be accessed here:


